# Benchmarking hyperparameter optimization strategies for crop genomic prediction

**DOI:** 10.3389/fgene.2026.1934071

**Published:** 2026-09-23

**Authors:** Huanping Xu, Yaoqi Wang, Yuting Ma, Hao Wang, Yongsheng Cao, Shen Yan, Jing Li, Yanjie Zhang, Yanqing Chen

**Affiliations:** 1 State Key Laboratory of Crop Gene Resources and Breeding, Institute of Crop Sciences, Chinese Academy of Agricultural Sciences, Beijing, China; 2 Research Institute of Pomology, Chinese Academy of Agricultural Sciences, Key Laboratory of Horticultural Crops Germplasm Resources Utilization, Ministry of Agriculture and Rural Affairs, Xingcheng, China; 3 Hebei Key Laboratory of Crop Stress Biology, College of Agronomy and Biotechnology, Hebei Normal University of Science and Technology, Qinhuangdao, Hebei, China; 4 Tobacco Research Institute, Chinese Academy of Agricultural Sciences, Qingdao, China

**Keywords:** crop breeding, genomic prediction, hyperparameter optimization, machine learning, plant genomics

## Abstract

Genomic prediction has become an important approach for accelerating crop breeding by using genome-wide marker information to predict complex traits. However, the performance of genomic prediction models is influenced not only by model selection but also by hyperparameter optimization strategies, which have received limited attention in crop genomic prediction studies. Based on four crop datasets, including maize, rice, wheat, and foxtail millet, we evaluated the prediction performance and computational efficiency of six representative genomic prediction models under four hyperparameter optimization strategies. The results showed that the optimal optimization strategy was jointly affected by crop species, target traits, and model types, and no single strategy consistently performed best across all scenarios. Bayesian optimization showed good prediction stability and achieved high prediction accuracy for most traits and models, especially in the rice and wheat datasets. Grid search and random search also showed strong competitiveness in some datasets. In contrast, although halving search reduced computational cost, it showed lower prediction stability in some cases. Our study highlights the important role of hyperparameter optimization in genomic prediction and provides a useful reference for selecting efficient optimization strategies in crop breeding applications.

## 1 Introduction

Complex traits in crops are typically controlled by multiple genes with small effects, together with environmental factors (Acquaah, 2012; Ewens, 1999; Yang et al., 2024). Traditional breeding relies heavily on phenotypic evaluation across multiple years and locations, which is time-consuming and costly. With the development of high-throughput genotyping technologies, genomic selection (GS) has become an important approach for predicting complex traits and improving selection efficiency in crop breeding (Jannink et al., 2010). GS uses genome-wide marker information to predict the breeding values or phenotypic performance of individuals, enabling the rapid selection of individuals with high genomic estimated breeding values (GEBVs) and greatly shortening the breeding cycle (Meuwissen et al., 2001). Many studies have demonstrated the potential of GS in crop breeding (Crossa et al., 2017; Wang et al., 2025; Chao et al., 2025; Ma et al., 2024). With the increasing scale of genotypic data, the continuous improvement of marker density, and the growing diversity of prediction models, the performance of GS models depends not only on the model itself but also on parameter settings (Alemu et al., 2024; Bayer et al., 2021). Different parameter combinations can lead to large differences in prediction accuracy and computational efficiency (Bischl et al., 2021; Liang et al., 2022a). Therefore, selecting an appropriate parameter optimization strategy is important for improving model prediction accuracy and reducing computational cost (Bayer et al., 2021; Crossa et al., 2025).

Commonly used methods in GS include traditional statistical models, machine learning models, and deep learning models. Traditional methods, such as ridge regression best linear unbiased prediction (rrBLUP), genomic best linear unbiased prediction (GBLUP), and Bayesian models, have been widely used in GS and show good stability and interpretability. Among these methods, rrBLUP uses an L2 regularization penalty to shrink genome-wide marker effects, which can reduce the risk of model overfitting and is suitable for predicting complex traits controlled by many genes (Endelman, 2011). GBLUP, proposed by VanRaden, uses a genomic relationship matrix instead of a traditional pedigree matrix to directly estimate genomic relationships and breeding values among individuals, showing good stability and reliability (VanRaden, 2008). With the development of machine learning techniques, several nonlinear models have also been introduced into GS studies, including random forest (RF) (Breiman, 2001), support vector regression (SVR) (Vapnik et al., 1996), extreme gradient boosting (XGBoost) (Chen and Guestrin, 2016), light gradient boosting machine (LightGBM) (Ke et al., 2017), and multilayer perceptron (MLP) (Rumelhart et al., 1986). Among these models, LightGBM is an efficient gradient boosting tree model with advantages such as fast training speed and low memory use, and has been widely applied to phenotypic prediction in different crops (Tong and Nikoloski, 2020). In recent years, deep learning models have also been gradually used in GS studies and have shown good potential for genomic prediction in crops such as rice, maize, and wheat, providing new method options for predicting complex traits (Jubair and Domaratzki, 2023; Montesinos-López et al., 2021).

However, the performance of different models is not consistent across crops, traits, and data scales, indicating that the performance of GS models is strongly data-dependent (Heslot et al., 2012; Azodi et al., 2019). For machine learning models in particular, prediction performance depends not only on the algorithm itself but also on hyperparameter settings, such as the number of trees, learning rate, and maximum depth in tree-based models, as well as kernel parameters and penalty coefficients in support vector regression (Bischl et al., 2021; Yu et al., 2020). Commonly used hyperparameter optimization strategies include grid search, random search, Bayesian optimization, and successive halving search, which differ in search space coverage, computational cost, and optimization performance. However, most crop genomic prediction studies have focused on comparing the performance of different models (Wang X. et al., 2018), while the suitability of different hyperparameter optimization strategies for GS models has received less attention.

In this study, we used four crop datasets with different sample sizes, including maize, rice, wheat, and foxtail millet, to compare the prediction accuracy and computational efficiency of six representative GS models (RF, XGBoost, SVR, LightGBM, MLP, and rrBLUP) under four hyperparameter search strategies (grid search, random search, Bayesian optimization, and successive halving search). We evaluated model performance in terms of prediction accuracy and computational efficiency across crop phenotypic prediction datasets of different scales and further assessed the effects of different search strategies on model improvement. Our results provide a reference for hyperparameter optimization and strategy selection in GS model development.

## 2 Methods

### 2.1 Dataset and preprocessing

Four crop datasets, including maize, rice, wheat, and foxtail millet, were selected to evaluate the performance of different GS models and hyperparameter optimization strategies across datasets with different sample sizes and genetic backgrounds. The basic information and preprocessing procedures for each dataset are described below.

The maize dataset included 8,652 F1 hybrids derived from 1,428 inbred lines. Field trials were conducted at five different ecological locations, and three agronomic traits were evaluated, including days to tasseling (DTT), plant height (PH), and ear weight (EW). SNP genotyping and genotype data preprocessing were performed according to the method described by Liu et al. (Liu et al., 2020). To obtain high-quality SNP markers, LD-based marker pruning was performed using the PLINK software package (Purcell et al., 2007). The sliding window size was set to 1 kb, with a step size of 100 SNPs, and SNPs with *r* ^2^ > 0.1 were removed. Finally, 32,519 SNPs were retained. Samples with missing values were then removed, leaving 8,439 F1 hybrids for analysis. To reduce data dimension and select markers with higher information content, the maximal information coefficient (MIC) between each SNP and the target trait was calculated using only the training set (Li et al., 2022; Kinney and Atwal, 2014). All SNPs were ranked according to their MIC values, and the top 10,000 SNPs were selected for subsequent model construction. For the test set, the corresponding SNPs were extracted based on the SNP indices selected from the training set to avoid information leakage from the test set (Supplementary Figures S1, S5).

The rice dataset was obtained from the 3 K Rice Genome Project (Wang W. et al., 2018). Five traits were selected for model evaluation, including culm length (Culm_length), days to heading in 2018HN (Days_to_heading_2018HN), grain length-width ratio (GLWR), plant height in 2018HN (Plant_height_2018HN), and thousand-grain weight (TGW). To reduce computational burden, the MIC method was also used to select the top 10,000 SNP markers. Samples with missing values were removed, and 2,799 rice accessions were retained for analysis (Supplementary Figures S2, S5).

The wheat dataset was obtained from the CIMMYT wheat germplasm collection and included 2,403 Iranian landraces of common wheat (*Triticum aestivum L.*) (Crossa et al., 2016). Genotype data were generated using DArT markers, with a total of 33,709 molecular markers. The presence and absence of alleles were coded as 1 and 0, respectively. The phenotypic traits included thousand-kernel weight (TKW), grain width (GW), grain hardness (GH), grain protein content (GP), and grain length (GL). Samples with missing values were removed, leaving 2,000 accessions for analysis. The MIC method was also used to select the top 10,000 markers for model construction (Supplementary Figures S3, S5).

The foxtail millet dataset included 680 sequenced germplasm accessions collected from 13 different geographic locations. In this study, straw weight data from five locations, including Anyang, Beijing, Changzhi, Dingxi, and Urumqi, were selected. Based on the results reported by He et al. (He et al., 2023), the large-effect SNP markers identified in their study were directly used as input features for model construction. After removing samples with missing values, 666 samples were retained for analysis. For each dataset, the samples were randomly divided into training and test sets at a ratio of 8:2. To ensure the repeatability of the experiments, a fixed random seed was used for data splitting. The same data split was applied to all models to avoid differences caused by different training-test partitions and to ensure a fair comparison of model performance. These previously identified markers were obtained independently from the present study and were directly used as predefined genomic features without any further phenotype-based selection using the current dataset, thereby avoiding information leakage from the current prediction task (Supplementary Figures S4, S5).

All phenotypic values used in this study were raw field observations and were not adjusted for environmental effects or converted into estimated breeding values. The original phenotype measurements were directly used as response variables for genomic prediction, and no additional environmental adjustment was applied before model training or evaluation.

### 2.2 Hyperparameter optimization strategies

Four complementary hyperparameter optimization strategies were selected because they represent distinct search principles and resource trade-offs. Grid search provides a deterministic exhaustive reference when the domain is small; random search is a simple stochastic comparator that can be more efficient when only a subset of dimensions is influential; Bayesian optimization uses previous evaluations to concentrate trials in promising regions; and successive halving tests whether early resource allocation and elimination can reduce computation. RF, XGBoost, SVR, LightGBM, and MLP were evaluated with all four strategies, whereas rrBLUP was retained as a fixed classical baseline. Each machine-learning model was also evaluated with its library default configuration on identical partitions so that the incremental benefit and computational cost of HPO could be measured against an untuned reference (Bischl et al., 2021; Bergstra and Bengio, 2012; Snoek et al., 2012; Jamieson and Talwalkar, 2015).

Hyperparameter search spaces were predefined for each machine-learning model and maintained consistently across all datasets to ensure a fair comparison among optimization strategies. The selected hyperparameters included parameters related to model complexity, regularization, and learning behavior. The detailed hyperparameter search spaces and candidate values for each model are provided in Supplementary Table S1. To enable a comparable evaluation of different optimization strategies, the search space for each model was designed as a compact discrete space with the same number of candidate combinations. Grid search, random search, Bayesian optimization, and successive halving search were applied under the same search space to evaluate their effects on model performance and computational efficiency. All search spaces were predefined before evaluation and remained consistent across different crops, traits, and datasets. RrBLUP was used as a classical baseline model with a fixed parameter setting and was not subjected to hyperparameter optimization. In addition, each machine-learning model was evaluated using its default configuration under identical data partitions to assess the contribution of hyperparameter optimization compared with untuned models.

### 2.3 Genotype data representation

SNP genotypes were represented using a 0/1/2 allele-dosage encoding. For each biallelic marker, the homozygous reference genotype was coded as 0, the heterozygous genotype as 1, and the homozygous alternative genotype as 2. The same encoding scheme was applied to all prediction models to provide a consistent numerical input representation and ensure fair comparisons among models and hyperparameter optimization strategies. Missing or invalid genotype values were handled using statistics estimated from the corresponding training fold only, thereby preventing information leakage from validation or test data.

### 2.4 Model construction

The six genomic prediction models were selected to represent different modeling paradigms, including linear statistical models, ensemble learning models, kernel-based methods, and neural networks. RrBLUP was included as a classical genomic prediction model and served as the baseline method. RF, XGBoost, and LightGBM represented tree-based ensemble learning approaches, SVR represented kernel-based nonlinear regression, and MLP represented neural network-based prediction. These models were selected based on their widespread applications in genomic selection, suitability for high-dimensional marker data, and complementary modeling characteristics (Endelman, 2011; Breiman, 2001; Rumelhart et al., 1986; Heslot et al., 2012). For machine learning models (RF, XGBoost, LightGBM, SVR, and MLP), both default configurations and optimized configurations obtained from four hyperparameter optimization strategies were evaluated (Chen and Guestrin, 2016) (Vapnik et al., 1996). The optimized hyperparameters and search spaces are described in Supplementary Table S1; Supplementary Data S1. RrBLUP was implemented as a fixed baseline model without hyperparameter optimization. All models were evaluated using identical training-test partitions and the same evaluation framework to ensure fair comparisons among genomic prediction models and hyperparameter optimization strategies.

### 2.5 Model performance evaluation

Prediction performance was evaluated using PCC, root mean squared error (RMSE), mean absolute error (MAE), and calibration intercept and slope. Outer-fold PCC values were summarized by their arithmetic mean, standard deviation, and a two-sided t-based 95% confidence interval, whereas pooled out-of-fold (OOF) PCC was computed after concatenating the five outer-validation predictions and was retained as the principal cross-validated generalization estimate. Independent-test PCC was the final generalization measure. Strategies were compared on matching outer folds using paired tests followed by Benjamini-Hochberg false discovery rate correction. Search-plus-refit time, candidate count, complete grid size, selected parameters, and the final parameter-source outer fold were recorded. HPO benefit was defined relative to the corresponding default model using paired changes in PCC and reductions in RMSE/MAE together with the additional computational cost.

### 2.6 Train-test split and nested cross-validation

The full dataset was first divided once into an 80% train_pool and a frozen 20% independent_test set using random_state = 42. Nested cross-validation was conducted exclusively within train_pool: five shuffled outer folds generated out-of-fold predictions, and a separate shuffled three-fold inner loop within each outer-training partition optimized mean Pearson correlation coefficient (PCC) (Kinney and Atwal, 2014; Galli et al., 2022). PCC was chosen as the search objective because genomic prediction primarily requires preservation of phenotypic ranking and linear association, while RMSE and MAE were retained as complementary error-scale measures. Preprocessing, including genotype validation, median imputation, optional mutual-information selection, and model-specific standardization, was refitted inside the relevant training fold. A search stopped after its predefined finite budget was exhausted (16 grid / random / Bayesian evaluations, or completion of the successive-halving schedule); MLP additionally used the estimator-level early-stopping rule described above. For each model-strategy combination, the parameter set associated with the highest inner three-fold mean PCC among the five outer folds was selected for final fitting; ties were resolved by choosing the lower-numbered outer fold. A complete preprocessing-and-model Pipeline was then refitted once on all of train_pool and saved before predicting train_pool and the independent_test set. The independent_test set was evaluated only once per final Pipeline and was never used for preprocessing fitting, hyperparameter selection, stopping, model selection, or parameter aggregation (Varma and Simon, 2006; Cawley and Talbot, 2010; Ziegel, 2003; Kaufman et al., 2011).

### 2.7 Wheat scalability experiment

Computational scalability was assessed using the wheat GH dataset by crossing four sample sizes (500, 1,000, 1,500, and 2,000) with four SNP counts (2,500, 5,000, 7,500, and 10,000), yielding 16 scale combinations. For each combination, samples and SNP markers were selected once using phenotype-independent permutations generated with random seed 42. No fixed 80:20 train-test split was applied in this scalability analysis. Each scale combination was evaluated using an outer-five/inner-three nested cross-validation design. The inner three-fold loop provided training-stage validation within each outer training partition, whereas predictions from the five outer validation folds were aggregated to estimate mean performance. Four models were evaluated using fixed, untuned settings: rrBLUP, implemented as Ridge regression with alpha = 1.0, Random Forest, XGBoost, and a multilayer perceptron. SVR and LightGBM were excluded. No hyperparameter optimization, including grid search, random search, Bayesian optimization, or successive halving, was performed, and SelectKBest was not applied because the sampled SNP count defined the final input dimension. Genotype validation, median imputation, and model-specific standardization were fitted using training data only within the corresponding cross-validation partitions. Scalability was summarized using the mean Pearson correlation coefficient (PCC), root mean squared error (RMSE), and model training time in seconds across the five outer folds.

## 3 Results

### 3.1 Model performance under different optimization strategies in maize

The independent-test results revealed a consistent advantage of the machine-learning models over the rrBLUP baseline in maize (Supplementary Figure S6). RrBLUP achieved Test PCC values of 0.80, 0.46, and 0.82 for days to tasseling (DTT), ear weight (EW), and plant height (PH), respectively. In contrast, the default machine-learning models reached 0.86–0.93 for DTT, 0.70–0.76 for EW, and 0.90–0.93 for PH. For all three traits, the 95% confidence intervals of the machine-learning estimates were visually separated from those of rrBLUP, supporting a clear performance difference in favor of machine learning under this split. Among the machine-learning models, however, the confidence intervals generally overlapped, so the small differences between their point estimates should not be interpreted as definitive pairwise superiority.

Hyperparameter optimization preserved this trait-dependent pattern (Figure 1; Supplementary Data S2–4). Across the four strategies, LightGBM yielded Test PCC values of 0.91–0.93 for DTT, 0.73–0.76 for EW, and 0.93 for PH; MLP yielded 0.89, 0.76–0.77, and 0.93–0.94; RF yielded 0.91, 0.73, and 0.92; SVR yielded 0.92–0.93, 0.74–0.77, and 0.94; and XGBoost yielded 0.93, 0.74–0.77, and 0.94, respectively. The 95% confidence intervals for Bayesian optimization, grid search, random search, and successive halving overlapped extensively within each model-trait combination. Thus, the figure does not support a robust statistical separation among optimization strategies, although successive halving produced slightly lower EW point estimates for LightGBM, SVR, and XGBoost. These interval-based comparisons are descriptive; formal claims of significance require the paired outer-fold tests specified in the evaluation protocol.

**FIGURE 1:**
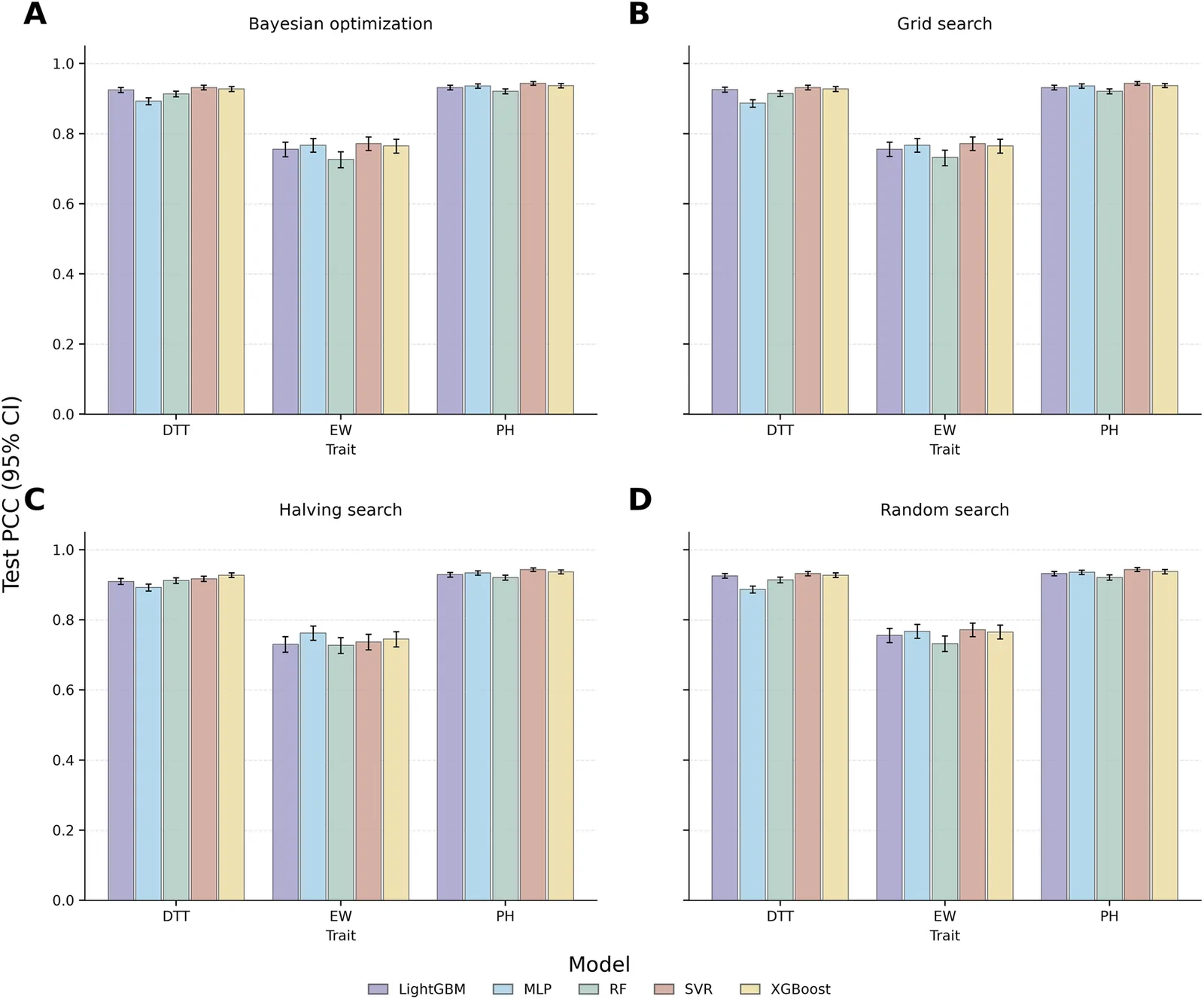
Independent test prediction performance of five machine learning models for maize traits under four hyperparameter optimization strategies. **(A)** Bayesian optimization; **(B)** grid search; **(C)** successive halving; and **(D)** random search. Within each panel, grouped bars represent the maize traits DTT, EW, and PH, while differently colored bars represent LightGBM, MLP, RF, SVR, and XGBoost. Bar heights indicate the Pearson correlation coefficient (PCC) obtained on the independent test set, and error bars indicate the corresponding 95% confidence intervals.

### 3.2 Model performance under different optimization strategies in rice

In rice, the default and baseline results showed stronger differences among models than among optimization strategies (Supplementary Figure S7). RrBLUP produced Test PCC values of 0.62–0.72 across the five traits. Default LightGBM and RF were consistently competitive (0.66–0.76 and 0.65–0.73, respectively), whereas default MLP was markedly weaker for days to heading, GLWR, and TGW (0.43, 0.12, and 0.31). The non-overlapping 95% confidence intervals for MLP on GLWR, relative to the other models, supported a clear performance deficit; for many comparisons among rrBLUP, LightGBM, RF, SVR, and XGBoost, the intervals overlapped and therefore did not establish clear pairwise separation. After optimization (Figure 2; Supplementary Data S2–4), SVR reached 0.71–0.78 across traits, including an increase in culm length from 0.56 under the default configuration to approximately 0.74, with little or no visual overlap between the corresponding 95% confidence intervals. LightGBM, RF, and XGBoost remained stable and competitive, whereas MLP remained substantially weaker for GLWR. Within most model-trait combinations, confidence intervals across the four search strategies overlapped, indicating that strategy-specific differences were generally small relative to uncertainty.

**FIGURE 2:**
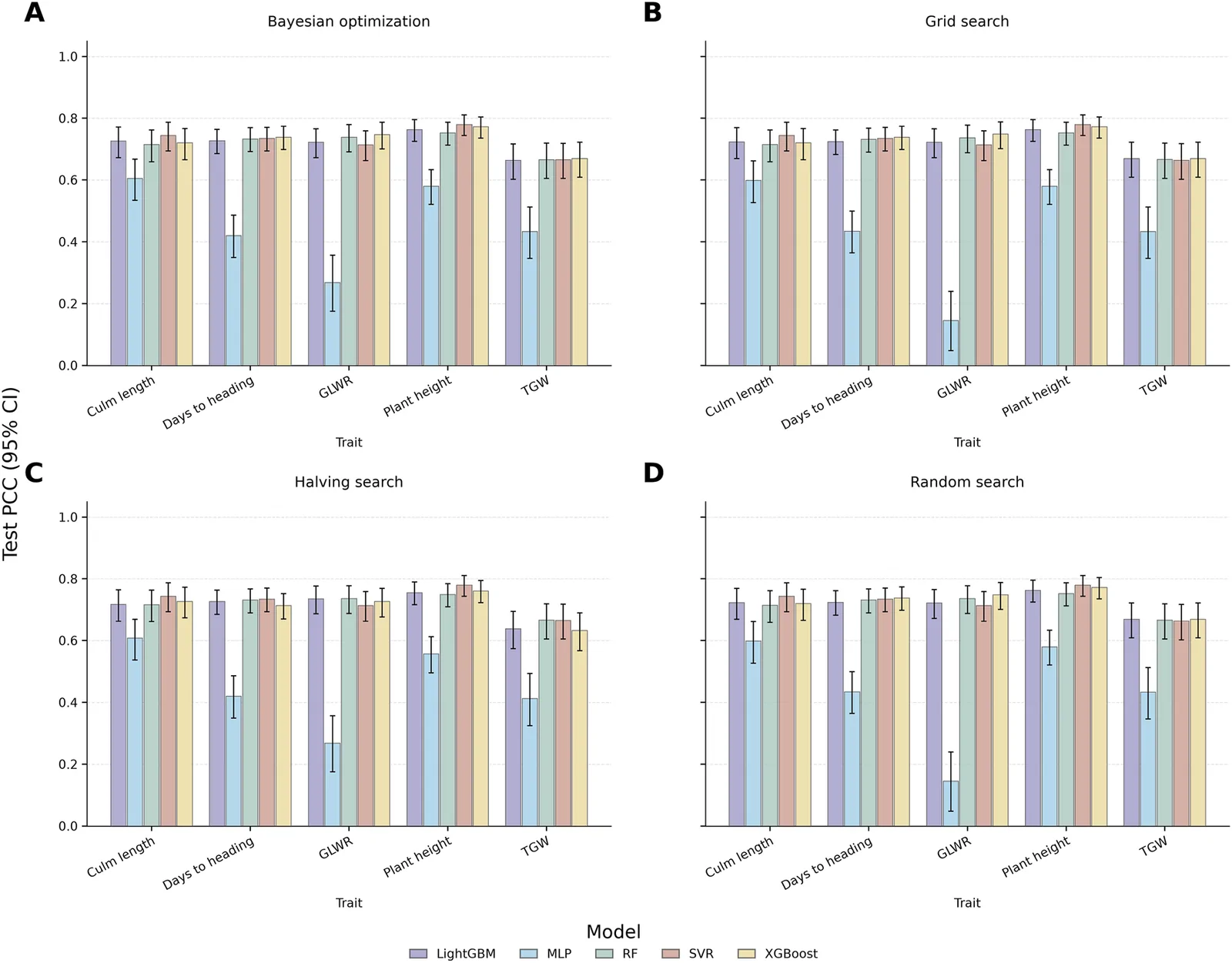
Independent test prediction performance of five machine learning models for rice traits under four hyperparameter optimization strategies. **(A)** Bayesian optimization; **(B)** grid search; **(C)** successive halving; and **(D)** random search. Within each panel, grouped bars represent culm length, days to heading, GLWR, plant height, and TGW, while differently colored bars represent LightGBM, MLP, RF, SVR, and XGBoost. Bar heights indicate the PCC obtained on the independent test set, and error bars indicate the corresponding 95% confidence intervals.

### 3.3 Model performance under different optimization strategies in wheat

For wheat, the default and baseline results revealed pronounced model-by-trait heterogeneity (Supplementary Figure S8). RrBLUP ranged from 0.47 for grain protein content (GP) to 0.72 for grain length (GL) and grain width (GW). Default LightGBM, RF, SVR, and XGBoost generally achieved moderate-to-high accuracy, whereas MLP was particularly weak for GP, GL, and GW (0.31, 0.34, and 0.13). For these traits, the MLP confidence intervals were visually separated from those of the strongest tree-based and kernel models, supporting a clear deficit; by contrast, the intervals for rrBLUP and several non-MLP models frequently overlapped, precluding a definitive ranking. Following optimization (Figure 3; Supplementary Data S2–4), LightGBM, RF, SVR, and XGBoost converged on similar Test PCC values for most traits, with the highest point estimates around 0.76–0.77 for GL and GW. The 95% confidence intervals across Bayesian optimization, grid search, random search, and successive halving overlapped broadly, so no search strategy showed consistent statistical superiority from the plotted intervals. MLP remained substantially less accurate for GP, GL, and GW despite tuning.

**FIGURE 3:**
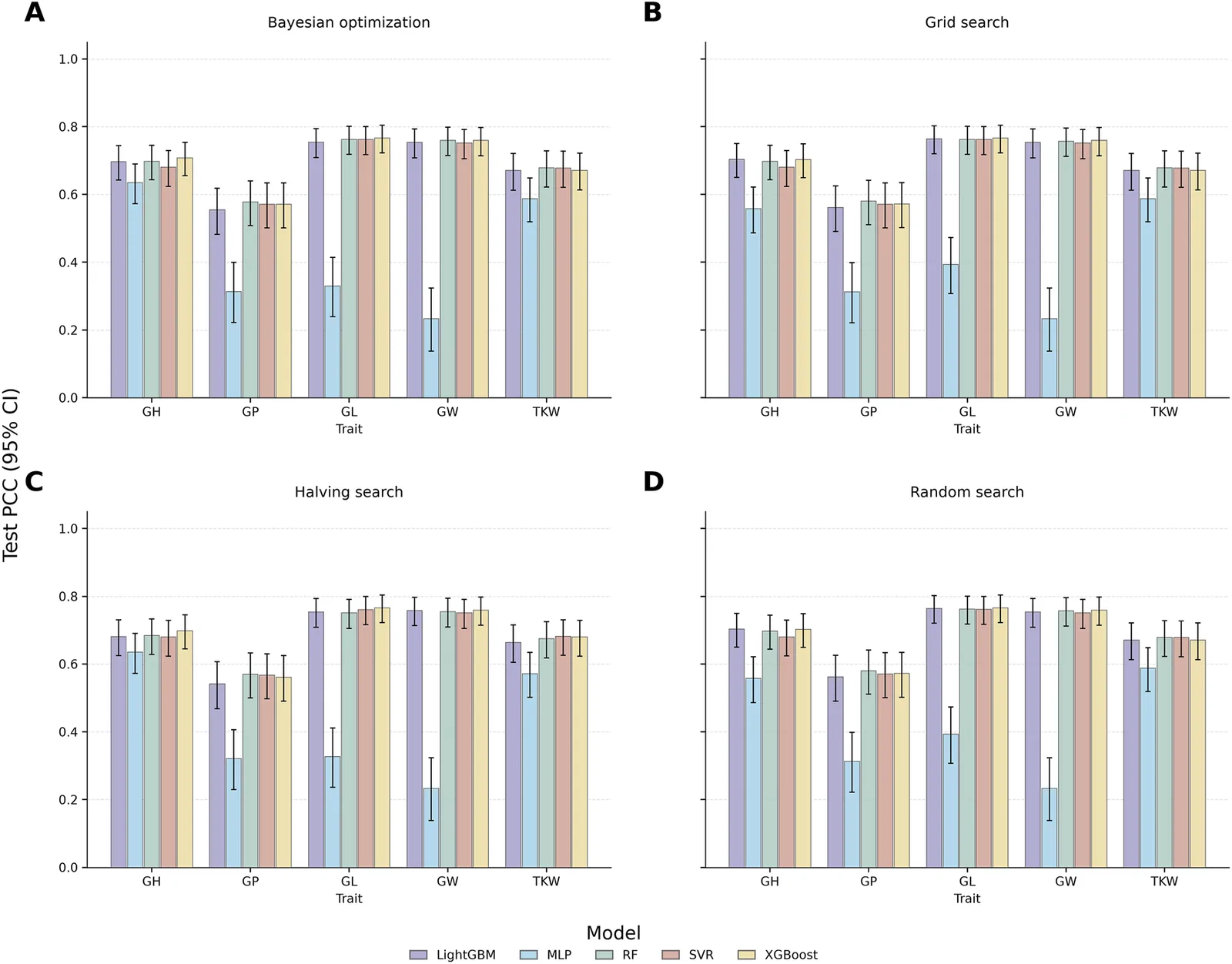
Independent test prediction performance of five machine learning models for wheat traits under four hyperparameter optimization strategies. **(A)** Bayesian optimization; **(B)** grid search; **(C)** successive halving; and **(D)** random search. Within each panel, grouped bars represent GH, GP, GL, GW, and TKW, while differently colored bars represent LightGBM, MLP, RF, SVR, and XGBoost. Bar heights indicate the PCC obtained on the independent test set, and error bars indicate the corresponding 95% confidence intervals.

### 3.4 Model performance under different optimization strategies in foxtail millet

Foxtail millet exhibited high prediction accuracy at several locations and strong model-by-location dependence (Supplementary Figure S9; Supplementary Data S2–4). The rrBLUP baseline ranged from 0.77 in Anyang to 0.89 in Changzhi. Among default models, MLP was broadly competitive (0.76–0.82), whereas XGBoost and RF showed lower point estimates at Anyang, Beijing, and Dingxi. Several default-model confidence intervals overlapped with the rrBLUP intervals, indicating that the apparent differences were not uniformly clear. Optimization substantially improved SVR and MLP (Figure 4): SVR reached 0.86–0.93 and MLP reached 0.86–0.91 across locations. In Anyang, the optimized SVR estimate (0.90) showed visually non-overlapping 95% confidence intervals with the rrBLUP baseline (0.77), supporting a clear improvement. At Changzhi and Urumqi, and for several other model-location comparisons, the intervals overlapped, so the higher point estimates cannot by themselves establish statistical superiority. Confidence intervals for the four search strategies were nearly coincident within most panels, providing no evidence of a universally superior optimization strategy.

**FIGURE 4:**
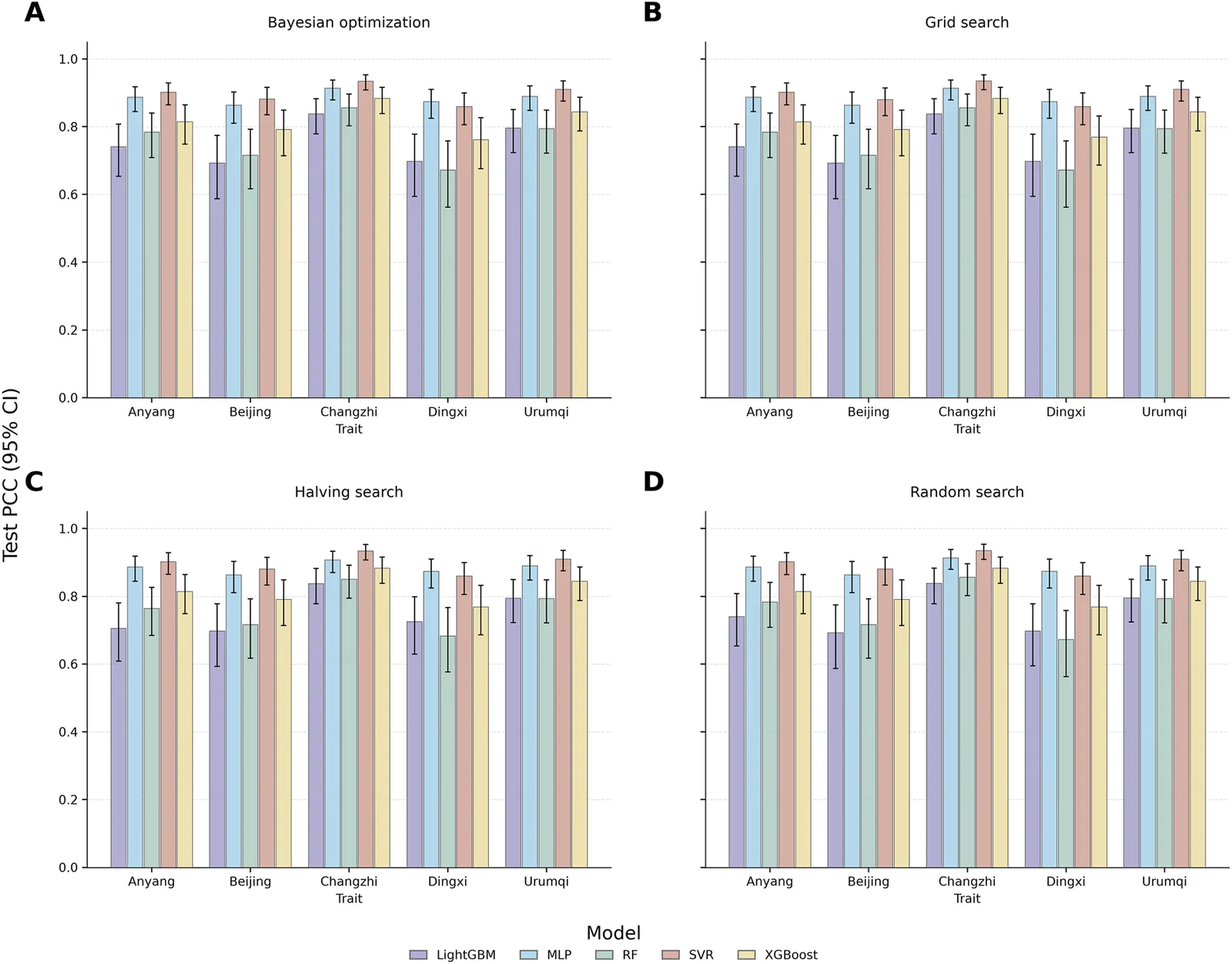
Independent test prediction performance of five machine learning models for foxtail millet across five locations under four hyperparameter optimization strategies. **(A)** Bayesian optimization; **(B)** grid search; **(C)** successive halving; and **(D)** random search. Within each panel, grouped bars represent Anyang, Beijing, Changzhi, Dingxi, and Urumqi, while differently colored bars represent LightGBM, MLP, RF, SVR, and XGBoost. Bar heights indicate the PCC obtained on the independent test set, and error bars indicate the corresponding 95% confidence intervals.

### 3.5 Computational efficiency comparison

Computational efficiency varied substantially by crop, model, and search strategy (Figure 5). RrBLUP was consistently the least expensive baseline. Among machine-learning models, default configurations and successive halving were generally the fastest, whereas Bayesian optimization was usually the most time-consuming. RF was the dominant computational bottleneck: its optimized runs extended to approximately 20,000–55,000 s in maize, 3,000–9,000 s in rice, 5,000–11,000 s in wheat, and roughly 60–230 s in foxtail millet. XGBoost, LightGBM, and SVR required substantially less time than optimized RF in most crops, while MLP showed intermediate costs. Absolute cross-crop times should be interpreted descriptively because dataset dimensions and computing environments may differ; the robust conclusion is the within-crop ordering of strategies and models.

**FIGURE 5:**
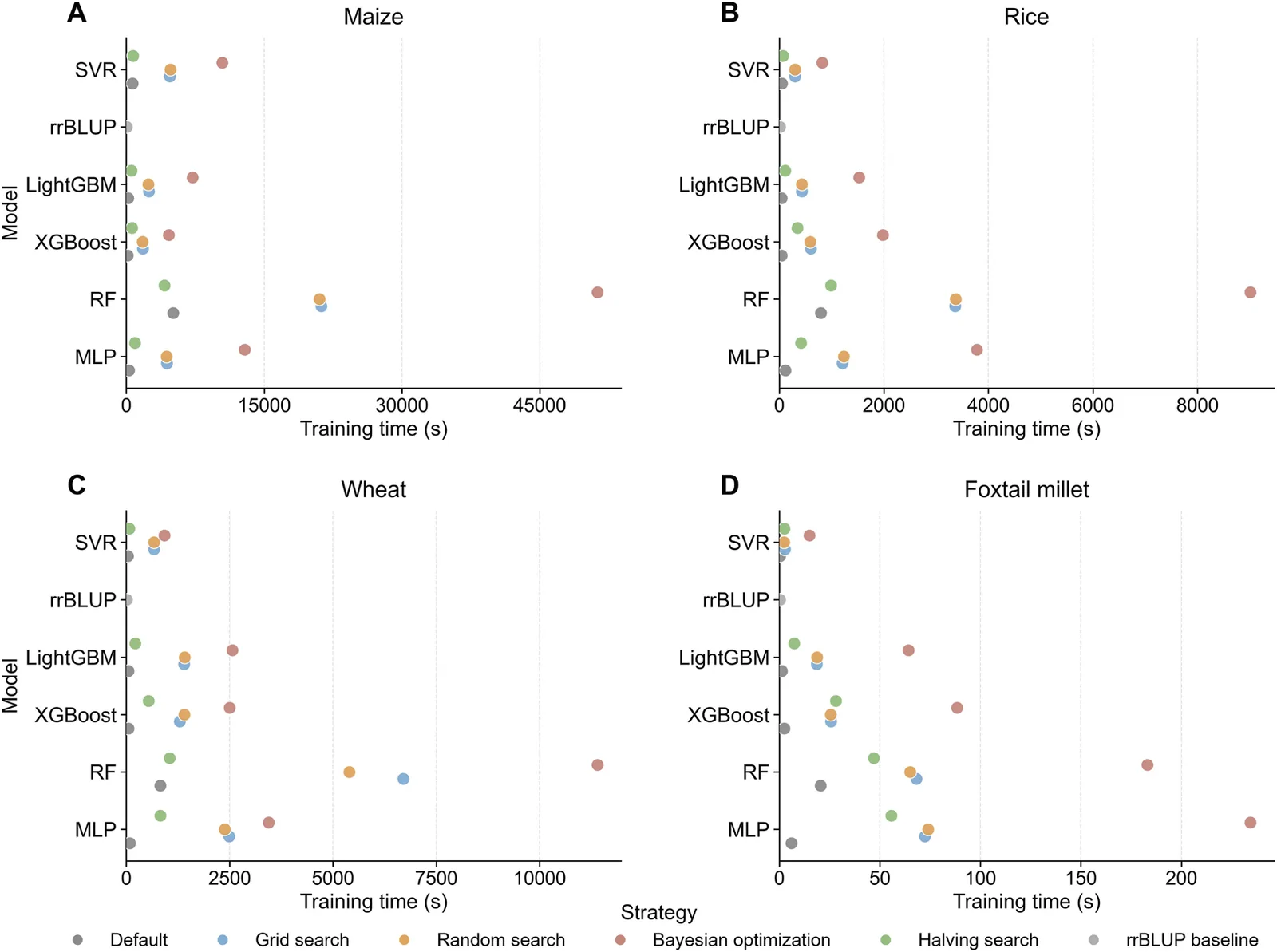
Computational efficiency across crop datasets. **(A)** Maize; **(B)** rice; **(C)** wheat; **(D)** foxtail millet. Points show training time for default, grid search, random search, Bayesian optimization, and successive halving; rrBLUP is shown as the baseline.

### 3.6 Wheat scalability experiment

The wheat scalability experiment demonstrated that computational cost increased with both sample size and SNP number, but at strongly model-dependent rates (Figure 6A). RrBLUP remained below a few seconds across the tested scales, whereas XGBoost, RF, and MLP increased from tens to more than 100 s per outer fold, with the largest marker sets and sample sizes incurring the highest costs. Prediction performance did not increase monotonically with scale (Figure 6B). XGBoost and RF were generally the strongest and stabilized at mean outer-fold PCC values of approximately 0.66–0.70 once the sample size reached about 1,000–1,500, whereas MLP improved from lower initial values but remained less accurate. RrBLUP was more variable and declined at the largest sample size for some SNP subsets.

**FIGURE 6:**
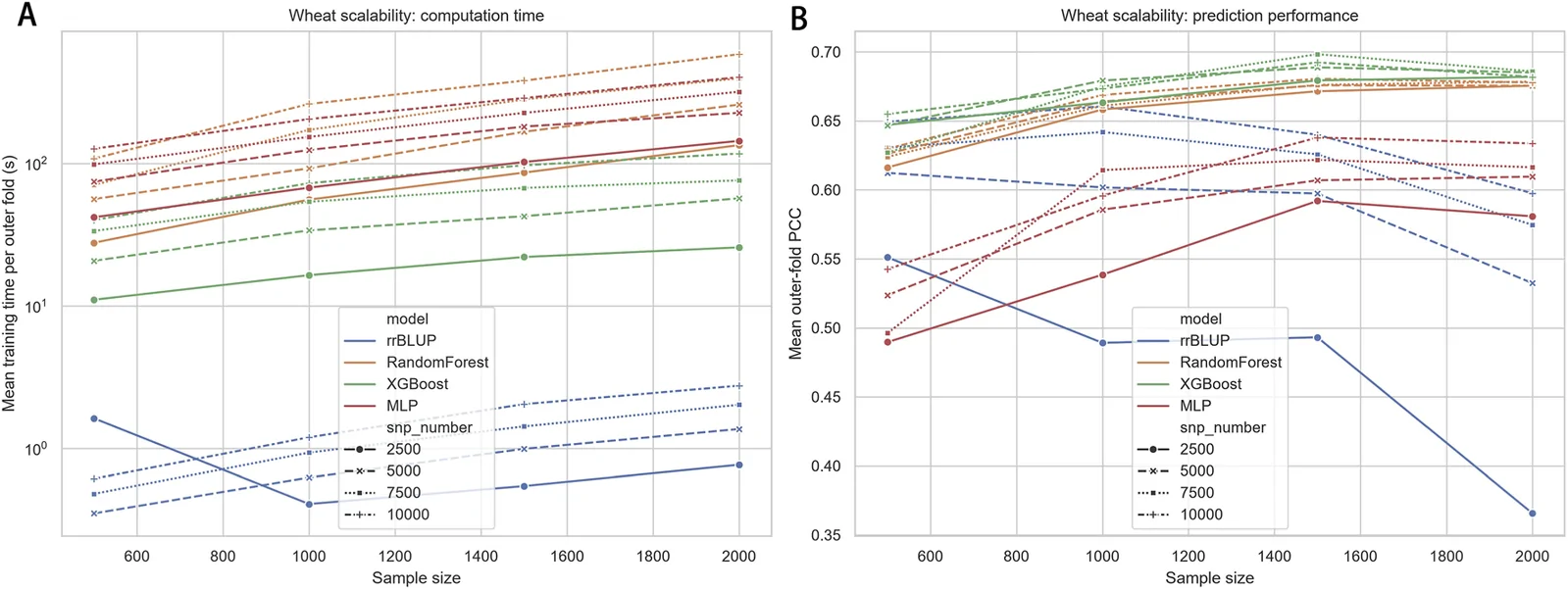
Wheat scalability experiment. **(A)** Mean training time per outer fold across sample sizes and SNP counts; the y-axis is logarithmic. **(B)** Mean outer-fold PCC across the same scale combinations. Colors denote models and line styles/markers denote SNP counts.

## 4 Discussion

The results delineate the conditions under which machine learning can improve on rrBLUP. Gains are most plausible when the training population is sufficiently large and representative, marker effects are heterogeneous, and the trait architecture contains dominance, epistasis, thresholds, or other nonlinear structure that can be exploited by kernels, trees, boosting, or neural networks. This pattern was clearest in maize, where all five machine-learning families exceeded rrBLUP for DTT, EW, and PH with visually separated 95% confidence intervals. Optimized SVR and MLP also improved prediction at several foxtail millet locations. In these settings, SVR can capture smooth nonlinear relationships in high-dimensional marker space; RF is robust to scaling and interactions; XGBoost and LightGBM efficiently model sparse nonlinear effects and higher-order interactions; and MLP offers the greatest representational flexibility when sample size and regularization are adequate.

Machine learning is not expected to dominate when genetic architecture is predominantly additive and infinitesimal, when the effective sample size is small relative to marker dimensionality, when phenotype quality or heritability is low, or when population structure and relatedness provide most of the predictable signal. Under these conditions, rrBLUP’s common shrinkage yields a stable, low-variance predictor and can match or outperform flexible models that overfit. The weak default MLP results for several rice and wheat traits illustrate the data demand and optimization sensitivity of neural networks. RF can be computationally expensive and may smooth numerous small additive effects; boosting methods require careful control of tree complexity, learning rate, and regularization; SVR depends on feature scaling and kernel parameters and can become costly as sample size grows; and MLP is sensitive to architecture, initialization, and early stopping. RrBLUP remains exceptionally fast, reproducible, and interpretable as an additive baseline, but its linear equal-shrinkage assumption limits its ability to capture non-additive architecture and heterogeneous marker effects.

The main contribution of this study is therefore not a claim that one algorithm or one search method is universally best, but a leakage-controlled comparative framework that jointly evaluates default and tuned models, uncertainty, computational cost, and scalability across crops and traits. A practical decision sequence follows from the results. Breeding programs should begin with rrBLUP as a mandatory low-cost benchmark; add RF, XGBoost, LightGBM, and SVR when nonlinear architecture is plausible and the training set is adequate; consider MLP only when sample size, regularization, and computational budget support it; and select hyperparameters exclusively within nested training folds. Compact grid or random search is appropriate for small discrete spaces, Bayesian optimization is most useful for broader or expensive continuous spaces, and successive halving is attractive when computation is constrained and early resource rankings are reliable. Final selection should balance out-of-fold PCC with error metrics, calibration, interval uncertainty, and training time, followed by one untouched-test evaluation. This framework directly converts the cross-crop comparison into model- and strategy-selection guidance rather than a simple leaderboard.

Several limitations constrain the scope of these conclusions. First, evaluation used one 80:20 random train-test partition. In maize, hybrids may share parents, while rice, wheat, and foxtail millet can contain family, kinship, population, geographic, or environmental structure (Galli et al., 2022; Liang et al., 2022b). Random splitting may therefore yield optimistic estimates and does not establish transfer to unrelated families or new environments. Future work should use repeated nested validation together with parent-, family-, kinship-, population-, and environment-grouped splits and external breeding cohorts. Second, mutual-information screening is a predictive filter rather than a QTN-mapping procedure. We did not compare multi-locus GWAS methods such as mrMLM, IIIVmrMLM, or Fast3VmrMLM, nor the multi-environment module of IIIVmrMLM for detecting main-effect QTN. These approaches should be evaluated as alternatives, but any phenotype-guided marker selection must be performed inside each training fold so that the independent test set remains untouched.

Finally, overlap of marginal 95% confidence intervals is only a descriptive diagnostic and is neither a formal test of no difference nor evidence of equivalence. The paired outer-fold comparisons remain the appropriate basis for inferential claims, preferably with multiplicity control and effect sizes. Training-time comparisons are also hardware- and implementation-dependent, and the wheat scaling experiment covered a finite range of sample and marker counts. Thus, the recommended workflow should be viewed as an evidence-based starting point that must be recalibrated for the target germplasm, trait architecture, deployment environment, and available computational resources.

## Data Availability

Publicly available datasets were analyzed in this study. This data can be found here: The raw whole-genome sequencing data of maize are available at NCBI under BioProject Accession No. PRJNA597703. Rice sequencing data are available through NCBI under project accession number PRJEB6180. The wheat dataset can be accessed at https://hdl.handle.net/11529/10548918. The foxtail millet dataset is available at https://www.cgris.net/millet.
