# Supplementary material for: Benchmarking hyperparameter optimization strategies for crop genomic prediction
Source: Front Genet. 2026 Sep 23;17:1934071. doi: 10.3389/fgene.2026.1934071 (PMC13641739; doi:10.3389/fgene.2026.1934071)
Supplement: Supplementary file 1 [file DataSheet2.docx]

**Benchmarking hyperparameter optimization strategies for crop genomic prediction**

Huanping Xu ^1,4, †^, Yaoqi Wang^2,†^, Yuting Ma^2^, Hao Wang^3^, Yongsheng Cao^1^, Shen Yan^1^, Jing Li^4^*, Yanjie Zhang^4^*, Yanqing Chen^1^*

1. State Key Laboratory of Crop Gene Resources and Breeding, Institute of Crop Sciences, Chinese Academy of Agricultural Sciences, Beijing 100081, China

2. Hebei Normal University of Science & Technology, Changli 066600, China

3. Tobacco Research Institute, Chinese Academy of Agricultural Sciences, Qingdao, 266101, China

4.Research Institute of Pomology, Chinese Academy of Agricultural Sciences, Key Laboratory of Horticultural Crops Germplasm Resources Utilization, Ministry of Agriculture and Rural Affairs, Xingcheng 125100, China

* Corresponding authors: Yanqing Chen, State Key Laboratory of Crop Gene Resources and Breeding, Institute of Crop Sciences, Chinese Academy of Agricultural Sciences, Beijing 100081, China. Email:chenyanqing@caas.cn; Yanjie Zhang, Research Institute of Pomology, Chinese Academy of Agricultural Sciences, Key Laboratory of Horticultural Crops Germplasm Resources Utilization, Ministry of Agriculture and Rural Affairs, Xingcheng 125100, China. Email: zhangyanjie@caas.cn; Jing Li, Research Institute of Pomology, Chinese Academy of Agricultural Sciences, Key Laboratory of Horticultural Crops Germplasm Resources Utilization, Ministry of Agriculture and Rural Affairs, Xingcheng 125100, China. Email: lijing@caas.cn.

**Supplementary Table S1** Hyperparameter search spaces used for model optimization in the genomic prediction experiments.

| **Model** | **Hyperparameter** | **Values tested / setting** |
| --- | --- | --- |
| Random forest | n_estimators | 200, 500 |
|  | max_depth | None, 15 |
|  | max_features | 'sqrt', 0.5 |
|  | min_samples_leaf | 1, 3 |
| XGBoost | n_estimators | 300, 600 |
|  | learning_rate | 0.03, 0.1 |
|  | max_depth | 3, 6 |
|  | subsample | 0.7, 1.0 |
| SVR | C | 0.1, 1.0, 10.0, 100.0 |
|  | gamma | 'scale', 0.001 |
|  | epsilon | 0.05, 0.1 |
| LightGBM | n_estimators | 300, 600 |
|  | learning_rate | 0.03, 0.1 |
|  | num_leaves | 31, 63 |
|  | max_depth | -1, 8 |
| MLP | hidden_layer_sizes | (64), (128), (128, 64), (256, 128) |
|  | alpha | 10⁻⁵, 10⁻³ |
|  | learning_rate_init | 10⁻⁴, 10⁻³ |
| rrBLUP | alpha | 1.0 (fixed baseline) |

*Note.* The same discrete search spaces were used for grid search, random search, Bayesian optimization, and successive halving. The common search budget was 16 candidate evaluations. rrBLUP was implemented as Ridge regression with alpha = 1.0 and was not subjected to hyperparameter optimization.

**Supplementary Table S2** Computational environment and software package versions.

| **Category** | **Software/package** | **Version** |
| --- | --- | --- |
| Programming language | Python | 3.14.4 |
| Numerical computation | NumPy | 2.5.1 |
| Data processing | pandas | 3.0.5 |
| Scientific computing | SciPy | 1.18.0 |
| Machine learning | scikit-learn | 1.9.0 |
| Gradient boosting | XGBoost | 3.3.0 |
| Gradient boosting | LightGBM | 4.7.0 |
| Bayesian optimization | Optuna | 4.9.0 |
| Parallel computing | joblib | 1.5.3 |
| Visualization | Matplotlib | 3.11.1 |
| Visualization | seaborn | 0.13.2 |
| Progress monitoring | tqdm | 4.70.0 |

**Supplementary Table S3** Model implementations and corresponding software versions.

| **Model** | **Implementation** | **Software** | **Version** |
| --- | --- | --- | --- |
| rrBLUP baseline | sklearn.linear_model.Ridge (alpha = 1.0) | scikit-learn | 1.9.0 |
| Random forest | sklearn.ensemble.RandomForestRegressor | scikit-learn | 1.9.0 |
| SVR | sklearn.svm.SVR (RBF kernel) | scikit-learn | 1.9.0 |
| MLP | sklearn.neural_network.MLPRegressor | scikit-learn | 1.9.0 |
| XGBoost | xgboost.XGBRegressor | XGBoost | 3.3.0 |
| LightGBM | lightgbm.LGBMRegressor | LightGBM | 4.7.0 |

*Note.* All versions were obtained from the Python virtual environment used to run the experiments. The rrBLUP baseline was operationalized as ridge regression, following the project implementation.

**Supplementary Table S4** Hyperparameter optimization protocol, stopping rules, randomization, and model-refitting workflow.

| **Item** | **Setting** | **Implementation details** |
| --- | --- | --- |
| Nested-CV design | Outer 5-fold; inner 3-fold | HPO was performed only within each outer-training fold; the frozen 20% independent test set was excluded. |
| Optimization objective | Maximize mean inner-CV PCC | The score was the mean Pearson correlation across the three inner validation folds. |
| Grid search | 16 combinations | All combinations in the discrete search space were evaluated in each outer fold. |
| Random search | 16 candidates | Because the budget equaled the 16-combination space, random search effectively evaluated the full grid. |
| Bayesian optimization | 16 Optuna TPE trials | TPESampler used its default density-ratio/expected-improvement mechanism; no custom acquisition function or pruner was specified. |
| Successive halving | 16 initial candidates; factor = 2 | Samples were the resource: candidates were halved as resources doubled from 30 samples until the resource limit or one candidate remained. |
| Stopping rules | Fixed budget or halving completion | Grid, random, and Bayesian searches stopped after 16 evaluations/trials. MLP additionally used estimator-level early stopping. |
| Random seeds | Base seed 42 | The split and outer CV used 42; inner folds and outer-fold tuning used 43-47; final refits used 42. |
| Outer-fold refit | Best candidate refitted on outer-training data | The selected configuration was fitted on the complete outer-training fold before outer-validation prediction. |
| Final selection and refit | Best inner mean PCC; full 80% train_pool | Ties were resolved by the earliest outer fold. All 26 Pipelines were independently refitted, saved, reloaded, and evaluated on the frozen test set. |

*Note. Settings were obtained from the executable workflow. The independent test set was excluded from HPO and parameter selection.*

**Supplementary Table S5** Computational hardware, parallelization, repetition, timing scope, and effective hyperparameter-search evaluations.

| **Item** | **Setting** | **Implementation details** |
| --- | --- | --- |
| Cloud processor | AMD EPYC 7Y43, 48 cores, 2.55 GHz | Cloud-computer configuration supplied by the user. |
| Installed memory | 32.0 GB RAM | Cloud-computer configuration supplied by the user. |
| Operating system | Windows 10 IoT Enterprise LTSC 21H2 | 64-bit x64 system; OS build 19044.4529. GPU acceleration was not used. |
| Parallelism | search_n_jobs = 16; final_model_n_jobs = 16 | Searches used 16 workers. Nested-CV RF/XGBoost/LightGBM estimators used one internal thread; their final refits used 16 threads. |
| Independent repetitions | 1 seed (42) | One 5 x 3 nested-CV run was performed; the five outer folds were paired folds, not repeated nested-CV runs. |
| Outer-fold timing | Search/fit wall-clock time | Included Pipeline preprocessing, inner-CV search, and outer-training refit; excluded prediction, metrics, file writing, and final-model training. |
| Final-refit timing | Full-train-pool Pipeline.fit | Excluded prediction, metrics, model saving/reloading, and independent-test evaluation. |
| Grid/random/Bayesian evaluations | 16 candidates or trials each | Each strategy required 48 inner-CV fits (16 x 3) plus one outer-training refit per model and outer fold. |
| Halving evaluations | 30 or 31 candidate-round evaluations | This corresponded to 90 or 93 inner-CV fits plus one refit; the stored candidate_count = 16 denotes initial candidates. |
| Final models | 26 refits per dataset | rrBLUP plus five models under default and four HPO strategies were independently refitted and tested. |

*Note. Hardware values describe the supplied cloud computer. Evaluation counts are per model and outer fold; halving counts include repeated candidate evaluations across rounds.*

Supplementary Figure 1. Distribution of phenotypic values between training and independent test sets for maize traits. Violin plots show the distributions of three maize traits, including days to tasseling (DTT), ear weight (EW), and plant height (PH), in the training and test sets.

Supplementary Figure 2. Distribution of phenotypic values between training and independent test sets for rice traits. Violin plots show the distributions of five rice traits, including culm length, days to heading, grain length-to-width ratio, plant height, and thousand-grain weight, in the training and test sets.

Supplementary Figure 3. Distribution of phenotypic values between training and independent test sets for wheat traits. Violin plots show the distributions of five wheat traits, including grain hardness (GH), grain protein content (GP), grain length (GL), grain width (GW), and thousand-kernel weight (TKW), in the training and test sets.

Supplementary Figure 4. Distribution of straw weight phenotypes between training and independent test sets across five foxtail millet locations. Violin plots show the distribution of straw weight values in the training and test sets for Anyang, Beijing, Changzhi, Dingxi, and Urumqi. The similarity of phenotype distributions between training and test sets indicates the representativeness of the data partition used for genomic prediction.

Supplementary Figure 5. Heritability estimates of evaluated traits across four crop datasets. Bar plots show the heritability values of traits used for genomic prediction in foxtail millet, maize, rice, and wheat datasets. For foxtail millet, heritability was calculated for straw weight across five locations; for maize, rice, and wheat, heritability values were calculated for individual traits.

Supplementary Figure 6. Independent test prediction performance of the rrBLUP baseline and the default configurations of five machine learning models for maize traits. Panels show rrBLUP (baseline), SVR, XGBoost, LightGBM, MLP, and RF. Bars represent Test PCC point estimates and error bars represent 95% confidence intervals for DTT, EW, and PH.

Supplementary Figure 7. Independent test prediction performance of the rrBLUP baseline and default machine learning configurations for rice traits. Bars show Test PCC point estimates and 95% confidence intervals.

Supplementary Figure 8. Independent test prediction performance of the rrBLUP baseline and default machine learning configurations for wheat traits. Bars show Test PCC point estimates and 95% confidence intervals.

Supplementary Figure 9. Independent test prediction performance of the rrBLUP baseline and default machine learning configurations across five foxtail millet locations. Bars show Test PCC point estimates and 95% confidence intervals.
